# A randomized, controlled pilot trial of the Emotional Faces Memory Task: a digital therapeutic for depression

**DOI:** 10.1038/s41746-018-0025-5

**Published:** 2018-06-06

**Authors:** Brian M. Iacoviello, James W. Murrough, Megan M. Hoch, Kathryn M. Huryk, Katherine A. Collins, Gary R. Cutter, Dan V. Iosifescu, Dennis S. Charney

**Affiliations:** 1 Click Therapeutics, Inc., New York, NY USA; 2 Department of Psychiatry, Mood and Anxiety Disorders Program, Icahn School of Medicine at Mount Sinai, New York, NY USA; 3 Fishberg Department of Neuroscience, Icahn School of Medicine at Mount Sinai, New York, NY USA; 4 Friedman Brain Institute, Icahn School of Medicine at Mount Sinai, New York, NY USA; 5 Department of Biostatistics, University of Alabama at Birmingham School of Public Health, Birmingham, AL USA; 6 Department of Psychiatry, NYU School of Medicine, New York, NY USA; 7 Nathan Kline Institute for Psychiatric Research, Orangeburg, NY USA; 8 Department of Pharmacology and Systems Therapeutics, Icahn School of Medicine at Mount Sinai, New York, NY USA

**Keywords:** Outcomes research, Therapeutics

## Abstract

There is an urgent need for more effective treatments for major depressive disorder (MDD). Digital therapeutics, such as computerized cognitive–emotional training interventions, represent a promising new strategy for treating MDD. Here we report a replication of efficacy of a digital cognitive–emotional training intervention designed to enhance cognitive control for emotional information-processing. In a randomized, double-blind, controlled study design, 51 participants with MDD in a current major depressive episode were randomly assigned to participate in a digital cognitive–emotional training regimen (Emotional Faces Memory Task (EFMT); *n* = 28) involving 18 sessions over 6 weeks, or an active control condition (CT; *n* = 23) involving computerized working memory training. MDD symptoms were assessed weekly using a clinician-rated measure (Hamilton Depression Rating Scale; Ham-D); and neurocognition (working memory), at baseline and study outcome. Mixed-effects model for repeated measures (MMRM) analysis of all participants randomized revealed a significantly greater reduction in MDD symptom severity (Ham-D) from baseline to outcome in the EFMT group (8.65 points) compared to the CT group (4.77 points) (*F*(6,205) = 3.23, *p* = .005, *d* = 0.46). Ten of 28 EFMT participants achieved clinical response (≥50% reduction in symptoms) compared to 4 of 23 in CT. Both groups exhibited similar, small improvements in working memory. This replicated the preliminary efficacy of a digital cognitive–emotional training approach for the treatment of MDD. EFMT may be a feasible and effective intervention strategy for MDD, but future studies to elucidate its mechanism of action are warranted. This study is registered with Clinicaltrials.gov (NCT: 01934491).

## Digital therapy: An effective intervention for depression

A computerized cognitive–emotional training program significantly reduces symptom severity in patients with Major Depressive Disorder (MDD). The Emotional Faces Memory Task (EFMT) in which patients are asked to identify and remember the emotions displayed in a series of faces, has been designed to activate brain regions involved in emotion processing and cognitive control simultaneously. A study led by Brian Iacoviello, Icahn School of Medicine at Mount Sinai, involving 51 non-medicated participants with MDD shows that after 6 weeks EFMT reduced both clinician-rated and self-reported measures of MDD symptoms. These findings suggest that digital therapies that can modulate activation patterns in brain areas affected in neuropsychiatric disorders hold great promise as a novel treatment approach.

## Introduction

There is an urgent public health need for more effective treatments for major depressive disorder (MDD), a disabling condition associated with significant morbidity, mortality and public health costs.^1,2^ Twenty to 30% of patients fail to achieve adequate therapeutic response to currently available treatments.^3,4^ To address the problems in treating MDD with currently available therapies, a recent National Institute of Mental Health Strategic Plan for Research^5^ calls for the development of novel interventions that focus upon known cognitive and behavioral correlates of depression, and suggests development of interventions that are portable and accessible to the general public and reduce development times, but that are also clinically validated. In line with this, digital interventions have begun to emerge for a variety of conditions including MDD and have shown promising indicators of efficacy.

An example of a digital intervention for MDD with preliminary validation is a cognitive–emotional training intervention for MDD: the Emotional Faces Memory Task (EFMT).^6,7^ EFMT was designed to simultaneously engage the dorsolateral prefrontal cortex (DLPFC) and amygdala, which subserve cognitive control and emotion regulation impairments commonly observed in MDD. To do so, EFMT combines working memory and facial affect identification tasks, which have been shown to elicit activity in the DLPFC^8^ and amygdala,^9^ respectively. A version of this task confirmed simultaneous activation of the DLPFC and amygdala in a sample of healthy volunteers,^10^ but the activation effects in MDD patients have not yet been confirmed.

An initial pilot study provided proof-of-concept for the efficacy of EFMT in MDD.^6^ In that study the EFMT intervention was administered to a sample of 21 MDD patients twice per week for 4 weeks, in line with previous cognitive training regimens reported in the literature.^11,12^ Half of the sample was randomly assigned to an active sham training task, which involved a straightforward working memory training (adaptive N-back task with shapes as the stimuli, instead of emotional faces) that would elicit DLPFC activation^8^ (but not amygdala as no emotion processing is occurring). The efficacy of EFMT was evaluated using a battery of measures to assess changes in cognitive biases and rumination, neurocognition/working memory, and MDD symptoms. At study outcome, EFMT had elicited significantly greater reductions in MDD symptoms and negative affective bias compared to the control group.^6^ Both groups, however, showed similar improvements in working memory, indicating that cognitive improvement alone was not driving the MDD symptom improvement. These findings suggested that cognitive–emotional training holds the promise of enhancing cognitive control for affective information processing and modulating the underlying neural networks that are involved in these processes. The current study was designed to replicate the initial pilot study findings in a larger sample that was powered to detect the expected symptom-improvement difference between EFMT and the sham-training group. A second, larger pilot was recommended by the funding agency (NIMH) as the initial study was considered a demonstration of feasibility and they wanted to see positive results in a larger pilot trial prior to funding a large-scale clinical trial. Thus, a second pilot study was conducted to investigate whether the initial pilot study results could be replicated, in a new sample of 51 MDD participants who enrolled in a randomized study of EFMT compared to a sham-control condition involving working memory training.

## Results

Between August 2013–April 2017, 55 currently un-medicated MDD participants signed consent to participate in the study, and study follow-up visits occurred through June 2017 until the sample of *n* > 50 was achieved. Four of these participants exited the study prior to commencing the training regimen and were not randomized, two due to scheduling conflicts and inability to commit to the study visit regimen, and two due to no longer meeting eligibility criteria to participate (symptom severity). The intention-to-treat (ITT) evaluation sample included the 51 MDD participants randomized, with 28 randomized to EFMT and 23 to CT. Three participants failed to complete at least two training sessions in week 1 and were discontinued from the protocol prior to the week 1 assessment. Forty-eight participants completed at least 1 week of training and were included in the modified intention-to-treat (m-ITT) analysis, 26 in EFMT and 22 in CT. Thirty-seven participants (73%) completed all study visits including the outcome assessments, 20 in the EFMT group (71%) and 17 in the CT group (77%). Table 1 provides the demographic and clinical characteristics of the study sample.

**Table 1:** Demographic and clinical characteristics of the study sample

All randomized participants		
	EFMT	CT
*n*	28	23
Age (years)	35.43 (10.66)	34.57 (11.72)
Gender (% female)	75.0%	60.9%
Race		
Asian	4	2
Hawaiian or Pacific islander	0	1
Black/African American	5	9
White/Caucasian	14	8
More than one race	4	2
Unknown/Unreported	1	1
Baseline MDD severity (Ham-D)	19.25 (2.55)	19.48 (2.64)
Current MDD episode duration (months)	18.61 (19.69)	11.04 (11.60)
Number of MDD episodes (lifetime)	3.19 (3.35)	3.61 (3.12)
Axis I comorbidities (current)	Dysthymia 14.29%	Dysthymia 4.35%
	Social phobia 10.71%	Social phobia 8.7%
	Generalized anxiety disorder 3.57%	Specific phobia 8.7%
	Body dysmorphic disorder 3.57%	

Modified ITT sample		
	EFMT	CT
*n*	26	22
Age (years)	35.46 (10.22)	33.68 (11.19)
Gender (% female)	76.9%	63.6%
Baseline depression severity (Ham-D)	19.35 (2.56)	19.23 (2.41)
Current MDD episode duration (months)	19.73 (19.99)	11.14 (11.87)
Number of MDD episodes (lifetime)	3.36 (3.43)	3.65 (3.22)

Values indicate *n*’s or mean (SD) unless otherwise indicated

### MDD symptom severity

**Fig. 1:**
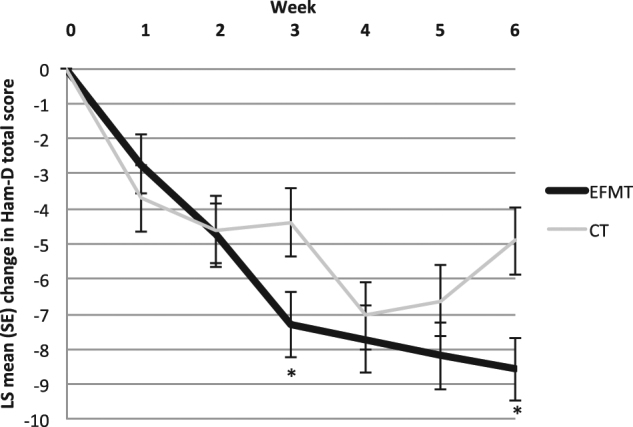
Change in depression severity over time. Least squares means change in Ham-D total score from baseline demonstrate superior reduction in the EFMT group compared to CT. Error bars indicate ±1 standard error of the mean. *p < 0.05 between groups

Mixed-effects model for repeated measures (MMRM) analysis of all 51 participants randomized revealed significant group × time interactions for change in HAM-D score (*F*(6, 205) = 3.23, *p* = 0.005, *d* = 0.46). Least squares means were tested between groups, with significant Ham-D mean differences at week 3 of 3.15 (se 1.34; *t*(205) = 2.36, *p* = 0.019, *d* = 0.23) and week 6 of 3.87 (se 1.32; *t*(205) = 2.93, *p* = 0.004, *d* = 0.30) demonstrating EFMT to have superior reduction in Ham-D score compared to the CT group. Table 2 provides the least squares means Ham-D score difference between EFMT and CT by week, along with the standard error, *t*-value, degrees of freedom, and corresponding *p*-value for the least squares means comparisons. Figure 1 depicts the least squares means change in Ham-D score from baseline over time for EFMT and CT groups. From baseline to outcome, EFMT participants showed a mean reduction of 44.94% in Ham-D score (from 19.25 to 10.60); CT participants showed a 24.49% mean reduction in Ham-D score (from 19.48 to 14.71). Ten of the 28 EMFT participants showed at least 50% reduction in Ham-D from baseline (meeting “clinical responder” criteria) while only 4 of the 23 CT participants achieved “responder” status.

**Table 2:** EFMT and CT Ham-D score difference in least square means by week

Week	Difference	Standard error	d*f*	*t*-value	*p*-value
Baseline	0.2283	1.1963	205	0.19	0.8489
Week 1	−0.7493	1.257	205	−0.60	0.5517
Week 2	0.3586	1.3246	205	0.27	0.7869
Week 3	3.1462	1.3355	205	2.36	0.0194
Week 4	0.8958	1.3596	205	0.66	0.5107
Week 5	1.7720	1.3737	205	1.29	0.1985
Week 6	3.8681	1.3181	205	2.93	0.0037

Sensitivity analysis in the m-ITT sample revealed a significant group × time effect on Ham-D score (*F*(1, 44) = 4.698, *p* = 0.036) indicating superior Ham-D reduction in the EFMT group compared to the CT group, controlling for baseline depression severity and the number of training sessions completed. These results were corroborated by the self-reported depression severity measure BDI-II, on which the EFMT group demonstrated a significantly greater reduction (31.28 (SD = 1.72) to 20.0 (2.28)) compared to CT (28.71 (1.77) to 25.29 (2.34)), *F*(1, 33) = 7.392, *p* = 0.011.

Post-hoc analyses investigated dose-response effects by analyzing the effect of the number of training sessions completed and the performance on the cognitive training task (mean N-level achieved across blocks in a session) in the m-ITT sample. The EFMT and CT groups did not demonstrate significantly different numbers of sessions completed (EFMT: 14.46 (SD = 5.27), CT: 14.91 (4.33); *t*(46) = 0.318, *p* = 0.752) or significantly different performance on the training task (mean N-level achieved; EFMT: 3.37 (1.73), CT: 4.40 (2.03); *t*(46) = 1.892, *p* = .065) although the CT group appeared to show an estimated greater performance on the CT task. There was a significant correlation between number of sessions completed and percent Ham-D response in the EFMT group (*r* = −0.497, *p* = 0.01) with more sessions completed associated with greater Ham-D response (a negative value) that was not present in the CT group (*r* = 0.152, *p* = 0.501). Performance on the training task was not significantly correlated with Ham-D response in either group (EFMT: *r* = −0.365, *p* = 0.066; CT: *r* = 0.406, *p* = 0.061) although the correlations were in opposite directions: greater performance was associated with greater Ham-D improvement in the EFMT group and lesser improvement in the CT group.

### Working memory effects

Both groups demonstrated average working memory capabilities at baseline (mean scaled score on WAIS subtests DSF, DSB and LNS) that were not significantly different between groups (10.79 (SD = 0.85) for CT, 11.00 (0.78) for EFMT; *t*(46) = 0.366, *p* = 0.716). Both groups demonstrated an improvement in working memory from baseline to outcome (outcome score: 12.01 (0.85) for CT, 11.63 (0.79) for EFMT), with a significant effect of time (*F*(1, 35) = 6.56, *p* = 0.015) but were not significantly different at outcome (*t*(35) = 0.399, *p* = 0.692). There was no significant group-by-time interaction (*F*(1, 35) = 0.798, *p* = 0.378) suggesting both groups working memory scores improved similarly during the course of the study.

### Symptom-level analysis

Analysis of baseline-to-outcome change in individual Ham-D symptoms in the m-ITT sample revealed significant group × time interactions for the following Ham-D items: item #2 (Rumination/feelings of guilt) (*F*(1, 46) = 4.462, *p* = 0.04)); item #7 (Work and activities) (*F*(1, 46) = 4.078, *p* = 0.049)); and item #11 (Somatic anxiety) (*F*(1, 46) = 6.11, *p* = 0.017)).

### Acceptability and perceived helpfulness of the interventions

At baseline, both EFMT and CT groups indicated that a cognitive training intervention “could be acceptable as a treatment for MDD,” with mean ratings just above 3 = “somewhat acceptable” (EFMT mean = 3.79 (0.98); CT = 3.81 (1.22); *t*(33) = 0.062, *p* = 0.951). When rating the acceptability of the intervention at outcome, both groups reported similar scores to baseline and again not significantly different between groups and relatively unchanged from baseline (EFMT mean = 3.94 (1.24); CT = 3.71 (0.91); *t*(28) = 0.555, *p* = 0.583).

At study outcome, EFMT and CT groups provided similar ratings for perceived helpfulness with attention and concentration (EFMT mean = 2.75 (1.13); CT = 3.14 (1.10); *t*(28) = 0.964, *p* = 0.343), perceived helpfulness with controlling thoughts (EFMT mean = 2.81 (1.17); CT = 3.14 (1.41); *t*(28) = 0.703, *p* = 0.488) and improving depressed mood (EFMT mean = 2.69 (1.01); CT = 2.86 (1.23); *t*(28) = 0.414, *p* = 0.682). The CT group reported greater perceived helpfulness with improving memory at outcome compared to EFMT (EFMT mean = 2.56 (1.21); CT = 3.79 (1.05); *t*(28) = 2.936, *p* = 0.007).

## Discussion

The current study replicated the results of our initial proof-of-concept study of the efficacy of a digital cognitive–emotional training intervention for MDD. Compared to an active working memory training control condition (CT), the EFMT group demonstrated greater MDD symptom reduction after completing a 6-week regimen of the training task, in both clinician-rated and self-report measures of MDD symptoms. A dose-response effect was also detected, with the number of training sessions showing a strong and significant correlation with symptom improvement in the EFMT group. The magnitude of MDD symptom response in both groups was similar to that observed in the previously published study^6^ (49% reduction in Ham-D score in study one vs. 45% in study two in the EFMT groups; 28% reduction vs. 25% in the CT groups). It is noteworthy that this is a second pilot study to yield a large effect size for EFMT, with this study revealing a greater than 3.8-point difference in Ham-D improvement between EFMT and CT. This difference would be described as clinically significant—a reduction of >2 points on the Ham-D is generally considered clinically significant (meaning the patient and clinician will typically note “improvement” when Ham-D scores have improved by 2 or more points); in addition, 2–4 point differences in Ham-D scores between groups are typically observed and interpreted as clinically significant in antidepressant treatment trials. However, the relatively small sample size still limits the generalizability of the current findings.

This study investigated changes in working memory capacity, and found that both EFMT and CT groups demonstrated similar, small improvements in working memory from baseline to outcome that were not significantly different between groups. Thus, the symptom improvement observed is not explained by improvement in cognition or working memory specifically. Both groups estimated cognitive training to be “somewhat acceptable” (3 on a 5-point Likert-scale) at baseline with no significant difference between groups and no significant change from baseline to outcome. At study outcome, both groups reported similar ratings for perceived helpfulness of the training intervention with improving attention, improving control over thinking and improving mood, with ratings in the “somewhat helpful” range (3 on a 5-point Likert-scale). The CT groups reported greater perceived helpfulness with improving memory compared to the EFMT group, even though both groups demonstrated similar improvements in working memory during the study period. This could be due to the fact that the CT group practiced a regimen of a working memory training task, whereas the dual task of emotion recognition and working memory in the EFMT task may have obscured the perceived benefit for memory training in that group.

The EFMT task aims to enhance cognitive control for emotional information processing and was designed to target abnormal activation patterns in DLPFC and amygdala. Given these neural targets, certain MDD symptoms would be expected to respond to the intervention, such as symptoms related to cognition and emotion, while others, such as neurovegetative symptoms, would not be expected to show quite as large of a response. For example, symptoms related to perseverative thinking (rumination and dwelling) and mood disturbance (chronic sad mood or anxious mood) might show more response than symptoms such as sleep and appetite disturbance. In line with this hypothesis, exploratory analysis of all 17 Ham-D symptoms, using a liberal threshold for significance of *p* < 0.05 not corrected for multiple comparisons, identified three symptoms that survived this threshold: rumination/guilt, motivation/activity, and somatic anxiety symptoms. These symptoms demonstrating a group-by-time interaction effect in favor of the EFMT group that are consistent with the mechanistic hypothesis of the cognitive–emotional training intervention, which targets cognitive control for emotional information-processing.

Taken together, these results support the continued evaluation of EFMT as an intervention for MDD, and support the development of training exercises designed to target cognitive–emotional processing abnormalities and the neural networks associated with these processes in mood and anxiety disorders. Other groups have reported on cognitive training interventions for depression: for example, computerized exercises that aim to enhance functioning of prefrontal cortical brain regions by use of a metacognitive exercise,^18^ or improve attention inhibition deficits in dysphoric individuals using a working memory training exercise.^19^ A recent meta-analysis of computerized cognitive training (CCT) intervention studies for depression suggests small to large effects for CCT on depressed mood, daily functioning, and certain cognitive domains (attention, working memory, and global functioning), suggesting that CCT may be an effective treatment option for adult depression.^20^ However, the mechanism by which this occurs is unclear due in part to treatments administered alongside CCT in several of the studies (participants were also treated with antidepressants, psychotherapy, or transcranial direct current stimulation during these trials). Further development and investigation is especially warranted as these types of exercises can be delivered remotely over a computer or smart device (phone, tablet) at the time and place of one’s choosing, and can therefore help to address the barrier to accessible and effective interventions that patients with depression too often encounter. Additionally, the combination of cognitive training interventions with traditional antidepressant medication treatment warrants investigation, as there could be significant synergy between these modes of treatment that could confer greater benefit to MDD patients than either treatment alone. Studies of cognitive training interventions in patients with only partial response to antidepressant medication treatment are also warranted to assess whether this could bring about full remission. In 2017 Pear Therapeutics had the first software-as-a-medical-device (SaMD) approved by the Food and Drug Administration as a class II medical device for treatment of a psychiatric disease. Other companies such as Akili Interactive Labs have been working to develop SaMD to treat ADHD and other conditions. Thus, the stage has been set for subsequent SaMD devices to be developed, evaluated and approved for the treatment of psychiatric disorders including MDD.

There are strengths and limitations associated with this study. Strengths of the study include the careful clinical characterization of the participant sample, randomization and blinding, and the experience of the investigators having previously completed a pilot study with the same methods. Finally, this study provides support for the efficacy of an intervention that has the potential to be disseminated on a large scale by leveraging electronic or digital means (e.g., web-based delivery; inclusion in a digital therapeutic platform), and ultimately reaching MDD patients that might not otherwise be able to access effective treatment. However, cognitive training studies have some serious limitations that have been highlighted previously in the literature.^21^ These include poorly-matched control conditions, short-lived results and exaggerated generalizability of findings.

There are limitations acknowledged in this study. First, the study relied on in-person visits to complete the training sessions and a relatively small sample size, which limits the generalizability of the current findings; future study of the feasibility of administering EFMT remotely in a “real world” setting, and in a large sample, is needed. Attrition in the current study was difficult to assess precisely due to the discontinuation criterion in the study protocol whereby participants were discontinued if they missed more than one session in any week or missed more than three sessions cumulatively. Still, 7 of 55 participants that signed consent discontinued the study by the end of the first week and were not included in the m-ITT sample, providing an estimate of expected attrition in similar studies in the future. An ITT analysis and secondary “completer” analysis were conducted to investigate potential non-attrition bias (participants that completed the treatment might be “special” compared to non-completers and confer exaggerated results); the consistency of results between the ITT and completer analyses suggests a minimal non-attrition bias in these results. Another limitation of the current study includes that it was conducted at a single site; future multi-center trials are indicated to adequately investigate the effectiveness of EFMT as an intervention for MDD. The lack of a matched passive comparison group means that the possibility that the effects observed are due to non-specific factors shared by both groups, including the behavior activation involved in study participation, can not be ruled out. In addition, the data collected in the present trial do not allow for investigation of the mechanism of action for this intervention. We hypothesize that the training confers functional connectivity changes between the DLPFC and limbic system, and that these changes are associated with symptom response. fMRI studies investigating this hypothesis are currently underway. Lastly, the middling ratings observed for acceptability of a cognitive training intervention as a treatment for MDD, which did not change after study completion despite significant symptom improvement in the EFMT group, may be seen as a potential limitation to the dissemination of these interventions in the future. However, the acceptability and perceived helpfulness measures employed in this study may not have been sensitive or sophisticated enough to elucidate actionable data regarding EFMT’s acceptability and perceived helpfulness. Nonetheless, patients’ motivation to adhere to a treatment regimen that is rated as “somewhat acceptable” and implemented remotely should be considered with caution when these interventions are disseminated on a large scale. Commercial efforts to enhance the acceptability and helpfulness of this type of intervention, for example by enhancing user engagement and other associated features, will be a focus of EFMT development in the future.

In summary, cognitive–emotional training exercises hold the promise of providing a novel paradigm for the treatment of MDD symptoms. A second study has now shown superior MDD symptom improvement associated with a cognitive–emotional training intervention compared to an active control condition. The magnitude of the effect was similar to a previously published pilot study, and the specific symptoms that appear to respond to the intervention are consistent with the hypothesized mechanism of action. Further study is warranted, in large samples and multiple sites, to confirm the effects of EFMT training, on specific symptom effects, and to explore mechanisms of action via neuroimaging the brain changes that are associated with EFMT.

## Methods

### Participants

Participants, recruited through advertisements in newspapers and online for depression research studies, were between the ages of 18–55 and evaluated by trained clinicians using the Structured Clinical Interview for DSM-IV-TR Axis I Disorders (SCID^13^). Participants met criteria for MDD diagnosis with a current episode. They could have other Axis I diagnoses (excluding psychotic disorders, bipolar disorders and substance abuse or dependence within the past 6 months) as long as their MDD diagnosis was primary. MDD severity, as measured by the Hamilton Depression Rating Scale—17-item version (Ham-D,^14^) had to be at least “moderate” (Ham-D ≥ 16). Participants with very severe MDD (Ham-D >27) were excluded and referred for treatment due to safety concerns surrounding participation in a placebo-controlled study and in which the treatment intervention had not yet been validated. Participants who had taken any antidepressant medications during their current MDD episode as well as those with a history of treatment non-response (2+ failures of an adequate trial of a standard antidepressant medication) were excluded from participation. Cognitive–behavioral therapy attendance in the 6 weeks prior to, or at any time during, the study, would also precipitate exclusion. Participants with visual or motor impairments that were thought to interfere with performance on the computerized exercise were also excluded. Reading ability at the 8th grade or above was required for enrollment to ensure that participants could comprehend all study assessments and questionnaires that were written at-or-below 8th grade reading level.

The protocol and study procedures were approved by the Program for the Protection of Human Subjects at the Icahn School of Medicine at Mount Sinai (ISMMS) and were conducted in accordance with the Declaration of Helsinki. After a pre-screening interview, eligible participants were informed about the study procedures and signed informed consent to complete screening and baseline procedures. Participants were aware that the study would evaluate the effects of two different memory-training exercises on memory and MDD symptoms. They did not, however, know the specific differences between the cognitive–emotional and control training (CT) paradigms, thereby maintaining the study blind. After completing the study, participants were debriefed, including a description of the study blinding involved and the rationale. Participants were reimbursed for each study session completed to compensate for time and travel expenses.

### Procedure

The study intervention was administered over 20 visits. At the first visit, the SCID and Ham-D were administered to confirm MDD diagnosis and determine symptom severity. A subsequent baseline evaluation assessed reading level, attention and working memory, MDD symptom severity, and participants’ perceived acceptability of a cognitive training exercise as an intervention for MDD. To measure reading ability, a Wide Range Achievement Test Reading test was administered. To assess attention span and working memory, a composite score was calculated as the mean scaled score from the Digit-Span Forward (DSF), Digit-Span Backward (DSB) and Letter-Number Sequencing (LNS) subtests of the Wechsler Adult Intelligence Scale-3rd Edition.^15^ MDD symptoms were evaluated using the clinician-rated Ham-D, and the self-report measure Beck Depression Inventory (BDI-II^16^). Acceptability of a cognitive training intervention as a treatment for MDD was assessed using a 5-point Likert-scale (“Using the scale below, please circle the number corresponding to how acceptable cognitive training would be to you, for the treatment of your depression symptoms, if cognitive training were a treatment option in the future.” 1: not at all acceptable; 3: somewhat acceptable; 5: very acceptable).

Participants were randomly assigned to the EFMT or CT groups by a research coordinator using a pre-determined randomization sequence for group assignment, with balancing in blocks of six participants in the sequence. Investigators and blinded study raters were kept blind to group assignment throughout the study. Participants were assigned to complete 18 training sessions over 6 weeks (approximately 20–35 min each, three times per week). Participants that failed to complete at least two sessions in any week, or that missed greater than three sessions during the course of the study, were discontinued as per the study protocol. Weekly Ham-D assessments were conducted by PhD or MD-level clinicians who were blind to group assignment. Ham-D raters were extensively trained to administer the assessment and demonstrated an ICC > 0.8 on two separate training interviews. An outcome evaluation was conducted within 1 week of completing the training sessions, at which time the baseline assessments were repeated. At the outcome evaluation, assessments of participants’ perceived acceptability of a cognitive-training intervention were again obtained, as were assessments of perceived helpfulness with improving memory, improving attention and concentration, improving control over thoughts, and improving mood. Assessments were obtained using a 5-point Likert-scale (1: not acceptable; 3: somewhat acceptable; 5: very acceptable).

### Cognitive training exercises

**Fig. 2:**
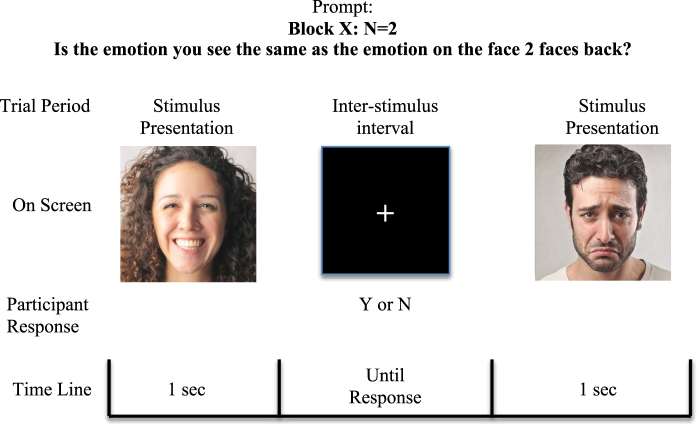
Example trial in the EFMT task. Participants observe an expression of facial affect shown on screen for 1 s and identify the emotion expressed. Participants then compare the observed emotion to the emotion observed N faces prior, in this case *N* = 2 faces prior. Copyright for the face images in the figure is owned by Adobe Systems Incorporated (http://stock.adobe.com)

The EFMT is a combination of emotion identification and working memory tasks, designed to simultaneously engage the DLPFC and amygdala, which subserve cognitive control and emotion regulation impairments commonly observed in MDD. To do so, EFMT combines working memory and facial affect identification tasks, which have been shown to elicit activity in the DLPFC^8^ and amygdala,^9^ respectively. In this task participants must identify the emotions they observe on a series of pictures of faces presented one at a time on a computer screen for 1 s, followed by a fixation cross for 1 s, and remember the sequence of emotions. Figure 2 depicts an example stimuli sequence in the EFMT task. Using an N-back working memory training paradigm, after each face observed participants are prompted to indicate whether the emotion on the face is the same as the emotion N faces prior. Each training session contains 15 blocks of the task during which the N level varies depending on the participant’s performance: the difficulty level increases or decreases across blocks as a participant’s accuracy improves or declines (respectively). The first training session begins with a difficulty level of *N* = 1 and the starting difficulty level for subsequent sessions is determined by performance at the prior session. The task is therefore tailored to a participant’s ability, ensuring a consistent challenge. N-back working memory tasks that are progressively challenging have been shown to improve working memory performance.^17^ EFMT utilizes a progressively challenging working memory paradigm and incorporates emotional stimuli throughout the task. The CT task is an active comparator to EFMT that consists of an identical cognitive-training paradigm, except that stimuli in the CT task are neutral shapes (circle, square, etc.). Because the CT task does not include emotional stimuli, no additional amygdala activation is induced. Thus the EFMT task involves exerting cognitive control over emotional information processing and the simultaneous activation of amygdala and DLPFC, whereas CT involves working memory and associated DLPFC activation only. The CT task in this study was the same that was utilized in the prior pilot study.

### Study design

The study was designed as a double-blind, randomized, controlled trial to replicate the effects of a regimen of cognitive–emotional training sessions on MDD symptoms and working memory in MDD participants. Random assignment to group (targeted to 1:1) utilized a randomization sequence, generated by a researcher not affiliated with this study, including random sequences in blocks of six participants to maintain group equivalence over time. The randomization scheme also accounted for completion of the training regimen, factoring this into the algorithm when assigning group status to aim for equivalent proportions of regimen completers in both groups. This was done due to a priori concern about unequal dropouts in favor of the control condition if participants suspected that the working memory training was not effective in addressing MDD. Randomization was implemented by a research coordinator at the initial cognitive training visit, who generated a unique login and password for each participant. This enabled participants to access the cognitive training exercise to which they had been assigned on a computer in the clinic at each visit. Research coordinators were not involved in any clinical ratings. All other study team members remained blind to group assignment.

### Data analytic strategy

The effect of EFMT vs. CT training on MDD symptom severity was the primary analysis of interest in this study. Primary analysis was conducted using a MMRM of all study participants randomized, with treatment group, visit, the interaction of treatment group and visit (used as a categorical variable to allow for characterizing the temporal effects without assuming linearity), and participant as a random effect, using an unstructured covariance matrix. Targeted sample size of 50 was calculated based on a power analysis for the above repeated measures analysis using G*Power 3.1 software, estimating a medium effect size, *α* = .05, power = 0.8, two groups, two measurements, correlation between measures = 0.5, and nonsphericity correction = 1. Sensitivity analyses were conducted in a m-ITT sample that included all participants who were randomized to either EFMT or CT, who completed at least one week of training and for whom at least 1 Ham-D score was obtained after the baseline assessment, with last observation carried forward (LOCF). This analysis included baseline Ham-D score and the number of training sessions completed as covariates. LOCF was utilized as a conservative means of addressing any missing Ham-D data in the m-ITT analysis. Self-reported depression severity was also investigated using repeated-measures analysis of BDI-II scores between groups. All data were checked to confirm assumptions required for statistical analyses conducted (e.g., normality, equal variances).

Secondary analyses aimed to evaluate changes in working memory and symptom-level effects on Ham-D items between groups. Repeated measures analysis at two time points (baseline and outcome) was conducted to explore for group or group-by-time effects on working memory (mean scaled score of WAIS subtests: DSF, DBS and LNS). Symptom-level analysis was conducted to explore whether specific symptoms respond to the EFMT vs. CT training. We hypothesized that certain symptoms associated with cognitive control abnormalities for emotion processing, such as sad mood, anxious mood, worry, and rumination/dwelling, would be responsive to the EFMT intervention, whereas other unrelated symptoms, such as sleep and appetite disturbance, would not. To explore this, repeated measures analysis of symptom change from baseline to outcome was conducted to investigate group-by-time interactions in all 17 Ham-D items. Secondary analyses also investigated differences between groups in perceived acceptability of the intervention at baseline, and perceived helpfulness of the intervention at study outcome on: depressed mood, attention and concentration, controlling thinking, and memory. These secondary analyses were conducted using t-tests between groups. Dose effects were investigated by analyzing correlations between the number of training sessions completed or the difficulty levels achieved, and Ham-D change. For these exploratory analyses, *p* < .05 was utilized as the critical alpha to identify target symptoms of interest for future investigation of this intervention, recognizing the multiple testing might increase our type I error.

### Data availability

The data reported in this manuscript are available from the corresponding author to facilitate replication. Access to the EFMT software can be granted to qualified researchers interested in viewing it for research purposes.
